# Study of the ultrastructure of *Enterococcus faecalis* and *Streptococcus mutans* incubated with salivary antimicrobial peptides

**DOI:** 10.1002/cre2.430

**Published:** 2021-05-05

**Authors:** Blanca Blancas, María de Lourdes Lanzagorta, Luis Felipe Jiménez‐Garcia, Reyna Lara, José Luis Molinari, Ana María Fernández

**Affiliations:** ^1^ Departamento de Microbiología y Parasitología, Facultad de Medicina Col. Universidad Nacional Autónoma de México Mexico City Mexico; ^2^ Instituto de Estudios Avanzados en Odontologia Dr. Yury Kuttler, Maestria en Endodoncia Mexico City Mexico; ^3^ Departamento de Biología Celular, Facultad de Ciencias UNAM, Col. Universidad Nacional Autónoma de México Mexico City Mexico; ^4^ Departamento de Bioquímica y Biología Estructural Instituto de Fisiología Celular, Col. Universidad Nacional Autónoma de México Mexico City Mexico; ^5^ Centro de Investigación en Ciencias de la Salud (CICSA), FCS Universidad Anáhuac México Campus Norte Huixquilucan Mexico

**Keywords:** Chromogranin A, Cystatin C, *Enterococcus faecalis*, Histatin 5, *Streptococcus mutans*

## Abstract

**Objectives:**

*Enterococcus faecalis* has been associated with root canal infections, while *Streptococcus mutans* has a central role in the etiology of *dental caries*. One of the main reasons of endodontic failure has been associated to the presence of *E. faecalis* and the formation of biofilms. *S. mutans* inhabits the oral cavity, specifically the dental plaque, which is a multispecies biofilm formed on the hard surfaces of the tooth. The biofilm formation is the main factor determining the pathogenicity of numerous bacteria. Natural antimicrobial peptides in the saliva protect against pathogenic bacteria and biofilms. The aim of this study was to assess the ultrastructural damage induced by salivary peptides in bacteria involved in biofilms has not been previously studied.

**Material and methods:**

*Enterococcus faecalis* and *S. mutans* incubated with cystatin C, chromogranin A, or histatin 5 were morphologically analyzed and counted. The ultrastructural damage was evaluated by transmission electron microscopy (TEM).

**Results:**

A decrease in bacterial numbers was observed after incubation with cystatin C, chromogranin A, or histatin 5, compared to the control group (*P* <  0.001). Ultrastructural damage in *E. faecalis* and *S. mutans* incubated with salivary peptides was found in the cell wall, plasma membrane with a decreased distance between the bilayers, a granular pattern in the cytoplasm, and pyknotic nucleoids.

**Conclusions:**

This study demonstrated that salivary peptides exert antibacterial activity and induce morphological damage on *E. faecalis* and *S. mutans*. Knowledge on the ultrastructural damage inflicted by salivary antimicrobial peptides on two important bacteria causing dental caries and root canal infections could aid the design of new therapeutic approaches to facilitate the elimination of these bacteria.

## 1 INTRODUCTION

*Streptococcus mutans* is considered the principal pathogen of dental caries (Takahashi & Nyvad, 2011). Dental caries is a disease that causes destruction of the mineralized tooth tissue (Koo, Falsetta, & Klein, 2013; Pitts et al., 2017; Wentao et al., 2018). Furthermore, dental caries is the most common cause of pulp infection. During the initial stage of caries, chronic inflammation occurs in the subodontoblastic region; infection by‐products, such as lipolysaccharides and lipoteicholic acid, spread to the periapex triggering immune responses that result in bone destruction induced by apical periodontitis (Silva et al., 2012; Stashenko, Teles, & D'Souza, 1998; Torabinejad, Eby, & Naidorf, 1985). *Enterococcus faecalis* is a Gram‐positive coccus and facultative anaerobe that is commonly found as a human intestinal commensal, but can be found temporarily in the oral cavity, here it has been associated with pathogenic oral manifestations like mucosal lesions in immunocompromised patients, (Wahlin & Holm, 1988), superinfecting organisms in periodontitis, (Rams, Feik, Young, Hammond, & Slots, 1992) and, most importantly, in persistent root canal infections (Hancock, Sigurdsson, Trope, & Moiseiwitsch, 2001; Pinheiro et al., 2003; Siqueira & Rôças, 2004). Enterococci can resist various intracanal treatment procedures, (Hancock et al., 2001; Sundqvist, Figdor, Persson, & Sjögren, 1998), this is attributed to the expression of different virulence factors, (Sedgley, Lennan, & Appelbe, 2005) enterococci invade dentinal tubules, (Portenier, Waltimo, & Haapasalo, 2003), to resist alkaline pH (Sedgley, Molander, & Flannagan, 2005), and possess the ability to form biofilms (Svensater & Bergenholtz, 2004). In fact, biofilms are the major cause for primary and secondary root canal infections and therefore, the success of endodontic treatment relies on their effective eradication (Nair, 2006). The National Institute of Health estimates that 60% of all human bacterial infections are due biofilms (Lewis, 2001). Biofilms are bacterial communities associated to surfaces and surrounded by an extracellular matrix (Donlan, 2001), that protects cells from antibiotics and immune cell attack Stewart (2002, 2015). Bacteria readily develop resistance to antimicrobials by several mechanisms, including mutations, activation of efflux pumps, and through the enzymatic inactivation of the antimicrobials (Munita & Arias, 2016). Bacteria that are not resistant to antibiotics can be tolerant by forming persistent biofilms, leading to chronic infections (Lebeaux, Ghigo, & Beloin, 2014). The World Health Organization (WHO) has considered that antibiotic resistance has become a critical public health concern that without coordinated proactive actions among all countries, by 2050, will cause more deaths than cancer (WHO, 2000). Antimicrobial peptides (AMPs) are promising candidates for the treatment of oral infections caused by biofilms, such as caries, periodontitis, implant‐associated infections and root canal infections (Lijun et al., 2011;Liu, Xu, Huo, Wei, & Ling, 2014; Mohamed, 2013; Hua et al., 2010).

Human saliva contains several antimicrobial polypeptides that play a vital role in the fight against invading pathogens, (Lamkin & Oppenheim, 1993), promote wound healing, (Oudhoff et al., 2008) and support apoptosis (Rudney, Staikov, and Johnson (2009)). Several proteins have been identified in all major glandular secretions, including secretory IgA, proline‐rich proteins (acidic, basic, and glycosylated families), amylase, statherins, histatins, lysozyme, lactoferrin, and lactoperoxidase, which form the key elements of the innate defense system in the oral cavity (Rudney et al., 2009; Azen & Oppenheim, 1973; Bennick & Connell, 1971; Eckersall & Beeley, 1971; Edgerton & Koshlukova, 2000; Hay, 1973; Hay & Oppenheim, 1974; Kauffman, Bennick, Blum, & Keller, 1991; Levine et al. (1987); Oppenheim, Hay, & Franzblau, 1971) Histatins (HST) are a group of neutral, basic proteins rich in the amino acid histidine (18–28 mol%) (Helmerhorst, Van't Hof, Veerman, Simoons, & Nieuw‐Amerongen, 1997; Lamkin, Migliari, Yao, Troxler, & Oppenheim, 2001; Lingstrom & Moynihan, 2003), found in human saliva at a concentration in the range of 5–100 μg/ml, comprising primarily of histatins 1, 3, and 5 at an 85% total composition, with histatins 3 and 5 being the most antimicrobially active in vitro (Oudhoff et al., 2008). Histatin 5 exhibits some activity against cariogenic bacteria in a multi‐species biofilm (Bobek & Levine, 1992). On the other hand, cystatins and mucins have been identified in submandibular/sublingual secretions (Lamkin et al., 2001; Bobek, Tsai, Biesbrock, & Levine, 1993; Bobek & Levine, 1992;Batoni, Maisetta, Brancatisano, Esin, & Campa, 2011). Cystatins are natural cysteine protease inhibitors that bind cysteine proteins, covering their catalytic region. Cystatin C has a molecular weight of 13,260 Da, constituted by 120 amino acids, without carbohydrates, and has two disulfide bridges located near the carboxyl terminal region. The cystatins present in saliva may protect oral tissues from degradation by cysteine proteases (Bobek & Levine, 1992; Oppenheim, Salih, Siqueira, Zhang, & Helmerhorst, 2007).

An antimicrobial peptide with remarkable antibacterial and antifungal activity is chromogranin A (CrA), which is the main member of the chromogranin family (Hagn, Schmid, Fischer‐Colbrie, & Winkler, 1986). Chromogranins are active against Gram‐positive and Gram‐negative bacteria and fungi and are not toxic for mammalian cells (Metz‐Boutigue, Garcia‐Sablone, Hogue‐Angeletti, & Aunis, 1993; Nolan, Trojanowski, & Hogue, 1985).

AMPs effectively kill bacteria, by multifactorial mechanisms, targeting different subpopulations in the biofilms. Moreover, they also can interrupt several stages of biofilm formation, including cell‐substrate adhesion and biofilm maturation, and can also kill bacteria in mature multi‐species biofilms promoting their detachment (Pletzer, Coleman, & Hancock, 2016). Furthermore, AMPs, have attracted much attention for their potential to be applied as antimicrobial agent, due their potent antimicrobial activities against a broad spectrum of microorganisms and also due their low bacterial resistance (Aoki, Kuroda, & Ueda, 2012; Guaní‐Guerra, Mendoza, Lugo‐Reyes, Santos‐SO, & Terán, 2010; Jenssen, Hamill, & Hancock, 2006; Parachin, Mulder, Viana, Dias, & Franco, 2012). Natural AMPs molecules are found in the oral cavity and exert antimicrobial activities against oral pathogenic bacteria and biofilms (da Silva et al., 2012). Despite the high microbial load in the oral cavity, abrasions, cuts, and minor surgical procedures rarely lead to infections, indicating that the host's defense mechanisms, including AMPs, are highly effective (Zasloff, 2002). These small cationic peptides also play relevant roles in the development of innate immunity and possess immunomodulatory functions (Auvynet and Rosenstein (2009); Lai & Gallo, 2009; Oudhoff et al., 2010; Steinstraesser, Kraneburg, Jacobsen, & Al‐Benna, 2011; Pasupuleti, Schmidtchen, & Malmsten, 2012;Choi, Chow, & Mookherjee, 2012). Because intracanal pathogens may evade chemomechanical debridement, (Figdor, Davies, and Sundqvist (2003), elimination of persistent pathogens and effective control of chronic immuno‐inflammatory responses have become crucial targets for the development of novel therapeutic initiatives in root canal therapy, potentially through the use AMP‐mimicking peptides (Lima et al., 2015). The use of AMPs as therapeutic alternatives and adjuvants is of continuous interest to combat dental caries (Mai et al., 2017). The effects of many natural or synthetic AMPs against cariogenic bacteria are being studied, including KSL, (Liu, Wang, Zhou, & Hu, 2011, L‐K6 (Shang et al., 2014), mPE (Beckloff et al., 2007), magainin 2 (McLean et al., 2014), MUC7 20‐mer (Altman et al., 2006), LFb 17–30 (Groenink, Walgreen‐Weterings, van ‘t, Veerman, & Amerongen, 1999), and Bac8c (Ding, Wang, & De Fan, 2014). Recently, a novel peptide antibiotic (Pro10‐1D) that potentially serve for the treatment of gram‐negative sepsis has been described (Krishnan, Choi, Jang, & Kim, 2020).

In the current study, we analyzed the ultrastructural damage in *E. faecalis* and *S. mutans* induced by salivary peptides on bacteria involved in the production of biofilms. We hypothesized that cystatin C, chromogranin A, or histatin 5 will effectively kill bacteria, allowing them to target different subpopulations of biofilms.

## 2 MATERIAL AND METHODS

### 2.1 Bacteria growth

*Enterococcus faecalis* ATCC 29212 and *S. mutans* strain ATCC 25175 were used in all the experiments. *S. mutans* was cultured under microaerophilic conditions with a candle jar at 37°C overnight in trypticase soy broth (TSB; Oxoid, Milan, Italy). *E. faecalis* was cultured under facultative anaerobiosis in brain heart infusion (BHI; BD Bioxon, Milan, Italy). Bacterial growth was monitored spectrophotometrically (Jenway Genova R0027, Fischer Scientific US**)** at 600 nm.

Ethical Approval was given by the Ethics committee of the Medicine Faculty UNAM with the reference number C54‐11.

### 2.2 Bacteria assays

*Enterococcus faecalis* and *S. mutans* were centrifuged twice with phosphate buffered saline at pH 5.2 (PBS, pH 5.2) and PBS, pH 7.2, respectively at 325 × *g* for 5 min. The supernatant was discarded, and the pellet was resuspended by vortexing in 1 ml PBS, pH 5.2, and 1 ml PBS, 7.2. The bacterial density was visually adjusted to a turbidity of 0.5 McFarland (1 × 10^8^ colony forming units (CFU/ml), diluted 1:10 with PBS, pH 7.2 *(E. faecalis)* and with pH 5.2 *(S. mutans)* and to yield 2 × 10^5^ CFU/ml.

### 2.3 Peptides

Lyophilized human histatin 5 and human chromogranin A WE‐14 were reconstituted with 1 ml of PBS, pH 7.0, containing 0.9% NaCl. *Cystatin C* human recombinant, expressed in *Pichia pastoris* was reconstituted with 1 ml of buffer Tris‐Base NaCl (pH 7.4) (Sigma Aldrich, St. Louis, MO).

### 2.4 Bacteria incubation with cystatin C, histatin C, or chromogranin A

For experimental and control group assays, 50 μl of *E. faecalis* and *S. mutans (*2 × 10^5^ CFU/per well) in PBS 7.2 was inoculated into 60 wells of microtiter plates containing the following:

*Enterococcus faecalis:*

1.‐ Control Group: 375 μl of PBS (pH 7.2).

2.‐ Experimental Group 1: 340 μl of PBS (pH 7.2) + 3.5 μg /35 μl of cystatin C.

3.‐Experimental Group 2: 373 μl of PBS (pH 7.2) + 2 μg (2 μl) of histatin 5.

4.‐ Experimental Group 3: 373 μl of PBS (pH 7.2) + 2 μg (2 μl) of chromogranin A.

*Streptococcus mutans:*

1.‐ Control Group: 375 μl of PBS (pH 7.2).

2.‐ Experimental Group 1: 340 μl of PBS (pH 7.2) + 3.5 μg/35 μl of cystatin C.

3.‐ Experimental Group 2: 373 μl of PBS (pH 7.2) + 2 μg (2 μl) of chromogranin A.

Growth inhibitory effects of the peptides in *E. faecalis* and *S. mutans* was determined by recording the colony forming units (CFU). Microtiter plates were incubated at 37°C, with shaking for 1 hr (Mackay, Denepitiya, Iacono, Krost, & Pollok, 1984). In order to evaluate the bacterial viability, 10 μl of the samples were diluted 1:1000, *S. mutans* and *E. faecalis* were cultured on Mueller Hinton Agar (BD Bioxon Milan, Italy) under microaerophilic, conditions and facultative anaerobiosis respectively, and incubated at 37°C during 24 hr. After that CFU/ml was determined.

### 2.5 Transmission electron microscopy

In order to determine changes in the ultrastructure of bacteria induced by the peptides, a suspension of 3 × 10^6^ CFU was incubated at 37°C during 24 hr, with 120 μg of cystatin C, histatin 5, or chromogranin A. After that, bacteria were washed three times with PBS, pH 5.2, at 4°C, rinsed in 0.15 M cacodylate buffer, and fixed in Karnovsky's solution during 1 hr at room temperature. After that, samples were transferred to 0.1 M cacodylate buffer, post‐fixed in 1% osmium tetroxide, dehydrated in ethanol [30,50, 70, 90, and 100%] and propylene oxide (1 hr), and embedded in Poly/Bed 812/DMP30 (Polysciences, Warrington, PA). Sections were then photographed with a Jeol JEM 1200 EXII transmission electron microscope (Fernández‐Presas et al., 2001.

### 2.6 Specimens analysis by electron microscopy

Changes in cell walls and plasma membranes of *E. faecalis* of *S. mutans* incubated with cystatin C, histatin 5, or chromogranin C and with cystatin C and chromogranin C, respectively, were evaluated using approximately 100 bacteria per condition at a magnification of 7500×. Data were obtained using the image processing package Fiji/ImageJ. The width of the bacterial cell wall and the distance between bilayers in the plasma membrane were determined.

### 2.7 Statistical analysis

Statistical analysis was performed using the Instat‐statistical software (GraphPad, San Diego, CA). Experimental and control conditions were compared using analysis of variance (ANOVA), followed by the Tukey–Kramer multiple comparisons. Significant differences were considered if the *P‐*value was <0.001.

## 3 RESULTS

### 3.1 Effect of histatin 5, cystatin C, or chromogranin A on the growth of *E. faecalis* and *S. mutans*

**FIGURE 1:**
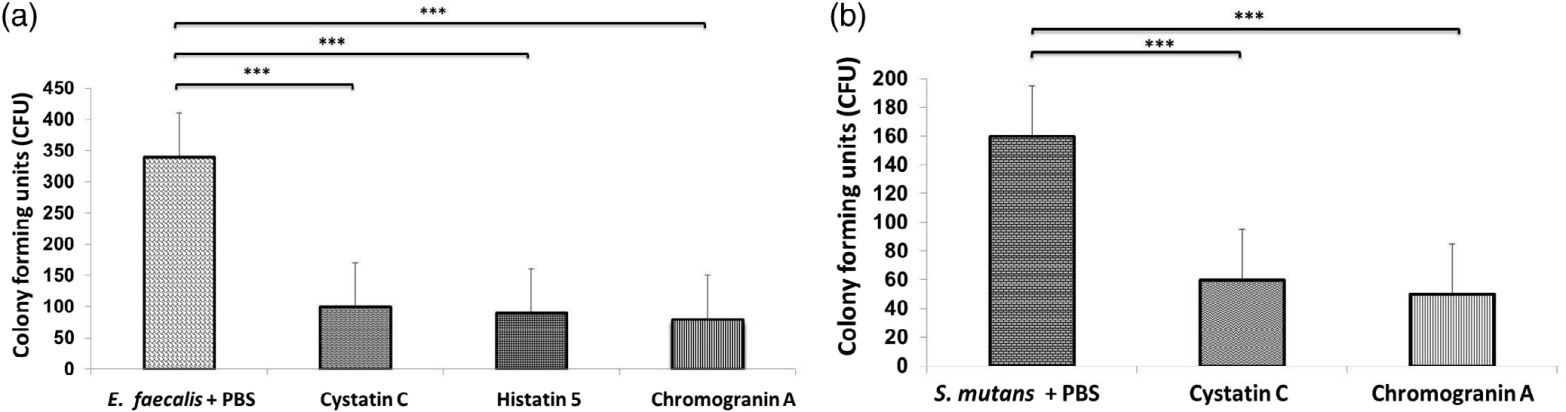
Bacteria incubated during 60 min with salivary peptides. (a) *Enterococcus faecalis* incubated with histatin 5, cystatin C or chromogranin A. (b) *Streptococcus mutans* incubated with cystatin C, or chromogranin A. The results are expressed as mean ± *SD* colony forming units (CFU) of *E. faecalis* and *S*. *mutans* statistically significant differences are expressed as ***P <  0.001 when compared to non‐treated control bacteria

The results from the effect of the different peptides on the growth of *E. faecalis* and *S. mutans* are shown as viable counts expressed in colony forming units (CFU). Statistically significant differences were found in the growth of *E. faecalis* and *S. mutans* incubated with the different peptides compared to bacteria incubated with PBS (Figure 1a, b) (*P* < .001).

### 3.2 Ultrastructural analysis of bacteria incubated with histatin C, cystatin C, or chromogranin A

**FIGURE 2:**
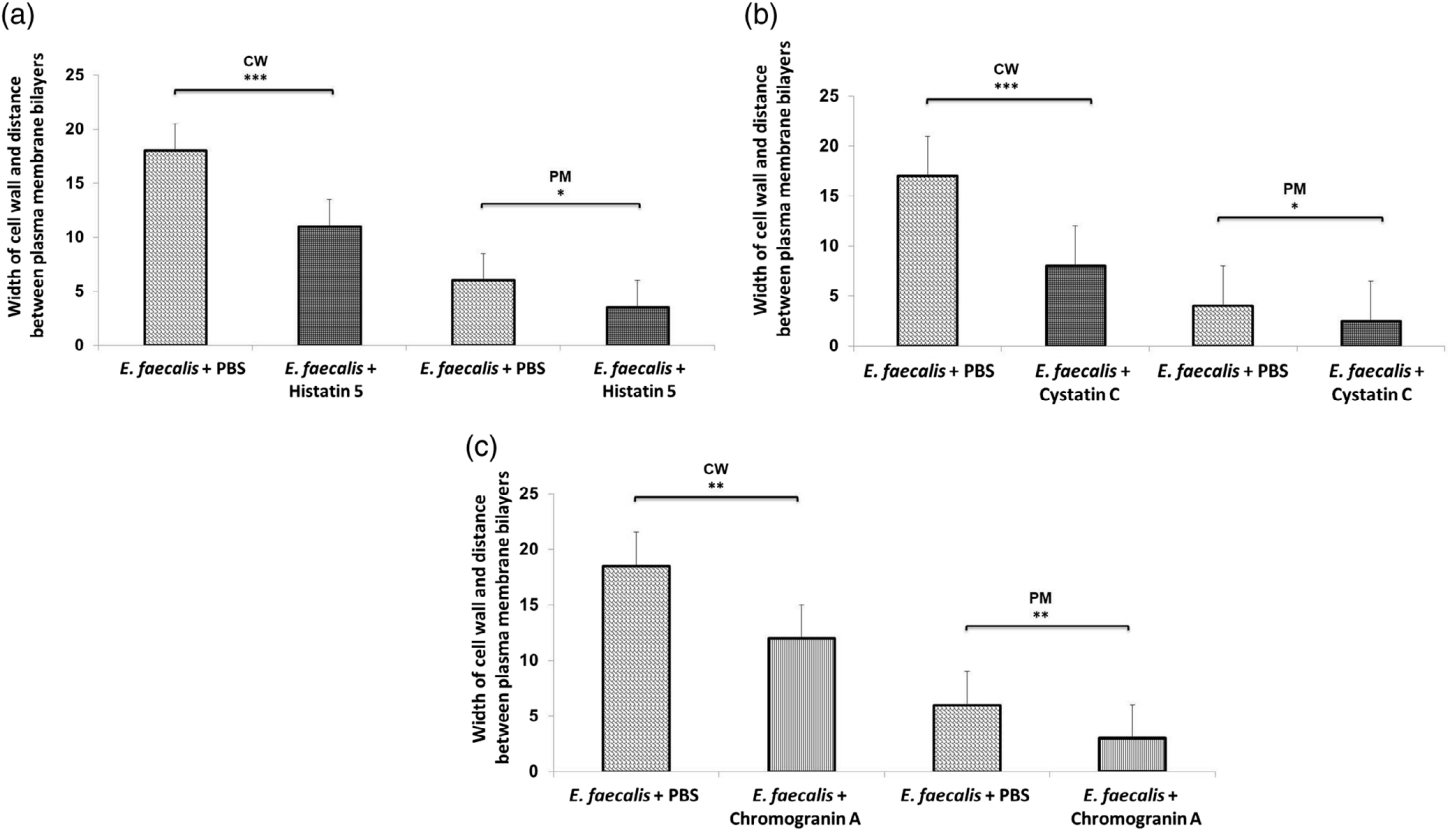
Mean width of the cell wall and the distance between plasma membrane bilayers of *Enterococcus faecalis* incubated with (a) Histatin, (b) Cystatin C, or (c) Chromogranin A.The bars show the mean ± *SD* of CW: cell wall, PM: plasma membrane. ** *P* <  0.001; *P <  0.01

**FIGURE 3:**
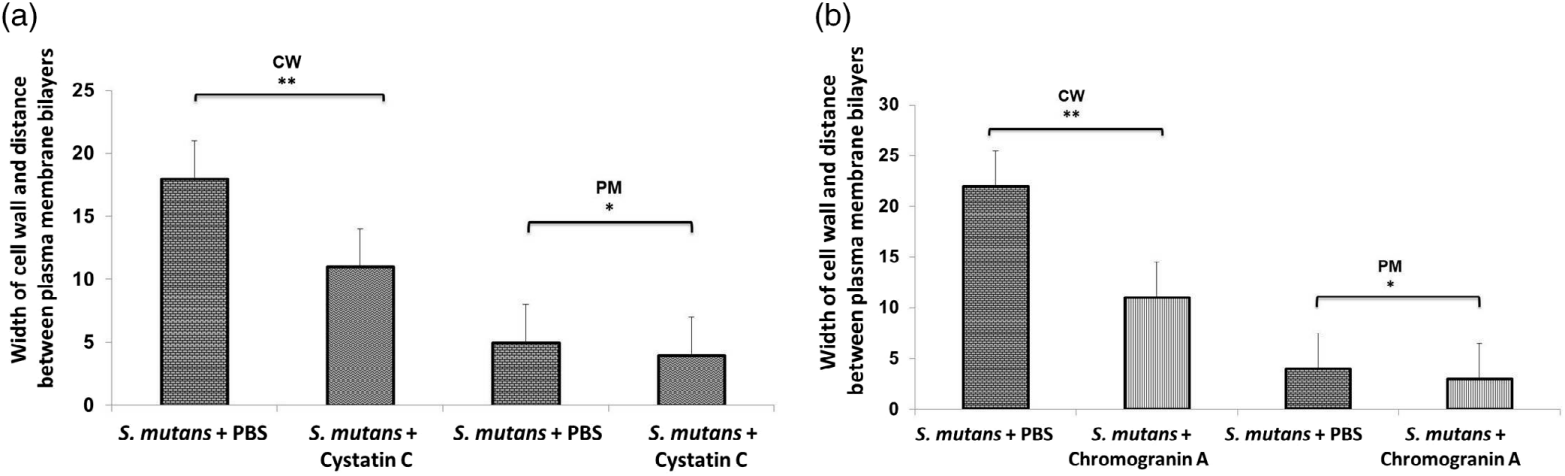
Mean width of the cell wall and the distance between plasma membrane bilayers of *Streptococcus mutans* incubated with (a) Cystatin C, (b) Chromogranin A. The bars show the mean ± *SD* of CW: cell wall, PM: plasma membrane. **P <0 .001; *P <0 .01

Transmission electron microscopy (TEM) was used in order to evaluate alterations induced in *E. faecalis* and *S. mutans* with different treatments. Differences in cell wall width and in the distance between the membrane bilayers were determined. Statistically significant differences in the cell wall width of *E. faecalis* incubated with histatin 5, cystatin C, or chromogranin A, compared to the untreated bacteria were found (*P* < 0 .001). The distance of the bilayers of the plasma membranes in the bacteria incubated with, histatin 5, cystatin C, (*P* <0 .01) or chromogranin A (*P* <  0.001) was significantly lower than the observed in the untreated bacteria (Figure 2a–c). In addition, the width of the cell wall of *S. mutans* incubated with cystatin C or chromogranin was significantly different that the observed in the untreated bacteria PBS (*P* <0 .001). The distance between the bilayers of the *S.mutans* incubated with the peptides was significant different compared with the control group (*P* < 0.01; Figure 3a, b).

*Enterococcus faecalis* and *S. mutans* incubated with the different peptides displayed extensive damage with detached cell walls, broken peptidoglycan and plasma membrane. A decrease in the plasma membrane bilayers was also observed. In contrast, no damage was observed in bacteria incubated with PBS.

**FIGURE 4:**
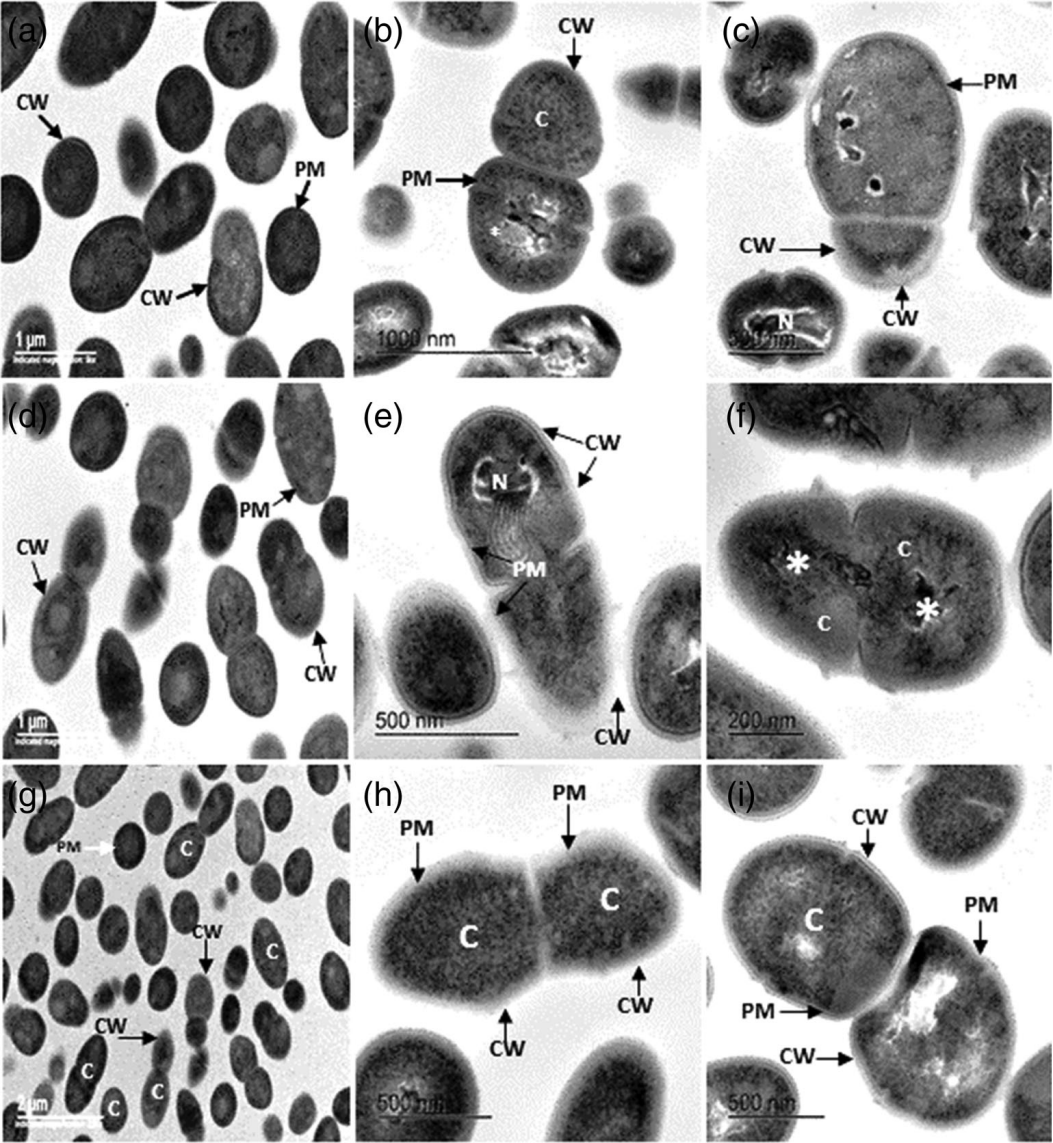
(a)–(i) Electron micrographs of *Enterococcus faecalis* incubated for 24 hr at 37°C with PBS, histatin 5, cystatin C, or chromogranin A. (a) Bacteria incubated in PBS, exhibit a homogeneous cytoplasm electron‐density (C), no damage in the cell wall (CW) and plasma membrane (PM) is observed. (b) and (c) *E. faecalis* incubated with histatin 5. (b) Note a granular pattern in the cytoplasm (C) and extrusion of the cytoplasmic content (*), discontinuities in the cell wall (CW) (arrows), and in the plasma membrane (PM) (arrows). (c) Some breaks in cell wall (CW) and plasma membrane (PM), are shown (arrows). Bacterial nucleoid is located eccentrically (N) with changes in its electron‐density. (d) *E. faecalis* incubated with PBS shows a well‐preserved cell wall (CW) (arrows) and plasma membrane (PM) (arrowhead). (e) Bacteria incubated with cystatin C, an eccentric nucleoid is observed (N), breaks in the cell wall (CW) and in the plasma membrane (PM) (arrows). (f) Chromatin condensation is exhibited (*), heterogeneous electron‐density is observed in some areas of the cytoplasm (C). (g) Bacteria incubated in PBS show a well‐preserved cell wall (CW) and plasma membrane (PM) and homogeneous electron‐density (C). (h) Bacteria incubated with chromogranin A, the cell wall peptidoglycan (CW) and the plasma membrane (PM) are not observed in some areas (arrows), and a granular pattern is observed in the cytoplasm (C). (i) Bacteria show a decrease in the cytoplasm electron‐density with extrusion of the cytoplasmic content (C), breaks in the cell wall (CW) and in the plasma membrane are observed

The main damage in the cell wall of bacteria incubated with the different peptides were interruptions in the peptidoglycan of the cell wall and a decrease in the width of the peptidoglycan and in the bilayers of the plasma membrane, compared to non‐treated bacteria. Electron microscopy analysis of *E. faecalis* incubated with histatin 5 showed interruptions in the cell wall and in the plasma membrane; in the cytoplasm, a granular pattern and a decreased electron density were observed (Figure 4b). The DNA was located eccentrically in bacteria with decreased electron‐density (Figure 4c). In contrast, control bacteria displayed intact cell walls, well‐defined membranes, and homogeneous cytoplasmic electron‐density (Figure 4a, d and g).

*Enterococcus faecalis* incubated with cystatin C showed cell wall infoldings and discontinuities in the peptidoglycan and in the plasma membrane. Bacterial DNA strands and decreased electron‐density were also observed (Figure 4e). In addition to the damage described in Figure 4e, bacteria exhibited a pyknotic nucleoid as shown in Figure 4f. A granular pattern in the cytoplasm with increased electron‐density was the most distinctive cytoplasmic feature observed in *E. faecalis* incubated with chromogranin A. Breaks in the cell wall and in the plasma, membrane are exhibited (Figure 4h). A decrease in the electron‐density of the cytoplasm was also observed (Figure 4i).

**FIGURE 5:**
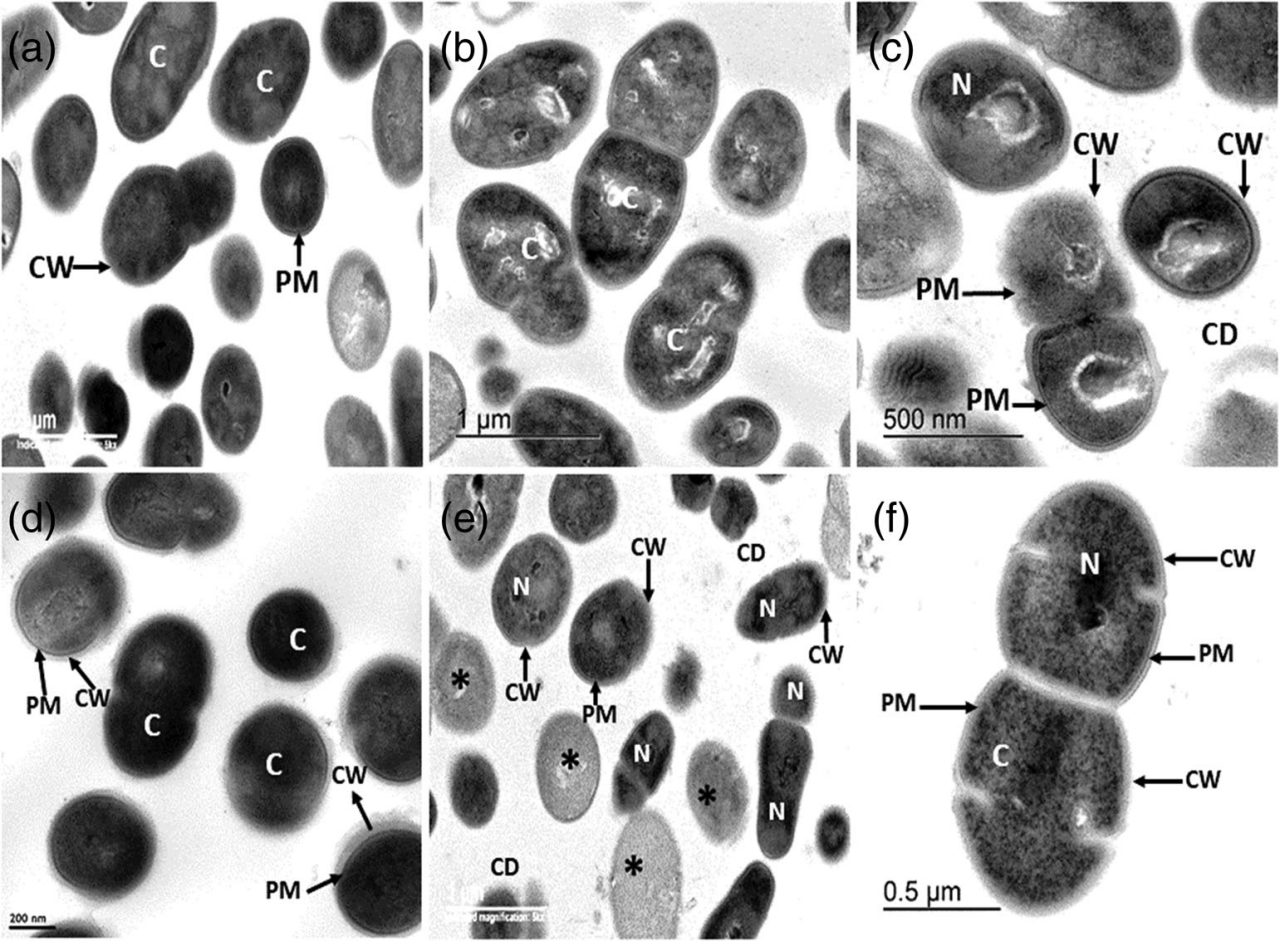
(a)–(f) Electron micrographs of *Streptococcus mutans* incubated for 24 hr at 37°C with PBS, cystatin C and chromogranin A. (a) Bacteria incubated in PBS, shown no damage in the cell wall (CW) and plasma membrane (PM), a homogeneous electron‐density in the cytoplasm was observed (C). (b) *S.mutans* incubated with cystatin C, shown decreased cytoplasm electro‐density and extrusion of the cytoplasm content (C). (c) the cell wall peptidoglycan (CW), and the plasma membrane (PM) is disrupted together, cellular debris (CD) is observed, condensation of the chromatin condensation is exhibited. (d) Bacteria incubated in PBS, showed intact cell walls (CW) (arrows) and well‐defined membranes. Cytoplasmic (C) and nucleoid (N) electron‐densities are homogeneous. (e) *S.mutans* incubated with chromogranin A, shows a condensed chromatin (N) interruptions in the cell wall (CW), and in the plasma membrane (PM), cellular debris (CD), and extensive morphological damage are evidenced in some bacteria (*). (f) Discontinuties in the cell wall (CW) and in the plasma membrane (PM), a granular pattern is exhibited in the cytoplasm (C), and pyknotic nucleoid (N)

*Streptococcus mutans* incubated with PBS displayed intact cell walls, well‐defined membranes, and homogeneous cytoplasmic electron density (Figure 5a). In contrast, *S. mutans* incubated with cystatin C displayed extensive damage with detached cell walls, breaks in the peptidoglycan and in the plasma membrane, and a decreased cytoplasm electron‐density (Figure 5b). The bacteria also showed pyknotic nucleoids, breaks in the plasma membrane, cellular debris, indicating cell lysis (Figure 5c). In contrast, untreated bacteria showed a normal cell shape with an undamaged cell wall structure and an intact plasma membrane, as shown in (Figure 5d).

*Streptococcus mutans* incubated with chromogranin A displayed extensive damage with detached cell walls, breaks in the peptidoglycan and in the plasma membrane, a granular pattern in the cytoplasm and a pyknotic nucleoid was observed (Figure. 5e, f).

## 4 DISCUSSION

In this study we analyzed the antimicrobial activity of cystatin C, histatin 5, or chromogranin A against *E. faecalis* and *S. mutans,* bacteria involved in the formation of biofilms. Biofilms are microbial communities that could adhere into abiotic surface, followed by their growth. It has been recognized that these bacterial species remain in a biofilm for a long time, and their formation plays an important role in infections (Mai et al., 2017). Bacteria organized in biofilms depict antimicrobial resistance, because their spatial organization hinders penetration of antimicrobial substances; the low growth rate and phenotypical modifications of bacteria in biofilms contribute also to this tolerance. Biofilms are extremely organized communities, in which interaction among cells confers tolerance, as previously shown by Kara, Luppens, and Ten Cate (2006). Biofilm formation is the main factor determining the pathogenicity of numerous bacteria, as occurs in *S. mutans* and *E. faecalis*, therefore, the inhibition of this process could become a potential method for the treatment of the dental caries **(**Krzyściak et al., 2015). Cationic peptides also have the potential to intercept multiple stages of biofilm formation, including prevention of cell substrate adhesion, destruction of biofilm architecture prior to stabilization, destruction of mature cell members in established communities, favoring the detachment of the biofilm (Pletzer et al., 2016).

The study of the ultrastructural damage in bacteria involved in the formation of biofilms, induced after their incubation with salivary peptides, showed extensive damage in different bacterial structures. This knowledge would allow for the design of compounds addressed at specific targets; for instance, we reported in this study that cystatin A, chromogranin A, and histatin 5 induce pyknotic nucleoids, a mechanism resembling an apoptosis‐like death in *E. feacalis* and in *S. mutans*.

We observed breaks in the cell wall and in the plasma membrane of *S. mutans* and *E. faecalis*, these breaks could allow the peptide to enter the bacterial cytoplasm causing extrusion of the cytoplasmic content, which could partially explain the death of the bacteria. In *E. faecalis* and *S. mutans,* as well as in other Gram‐positive bacteria, the primary interrelation of the peptide would occur between anionic peptides and teichoic acid. After this primary interaction, the peptides bind to the cell surface allowing their subsequent binding, which enables them to pass through the external membranes (teichoic and lipoteichoic acids) and interact with the bacterial plasma membrane (Brogden, 2005). Different mechanisms have been proposed, such as the perpendicular orientation of the peptide followed by penetration into the cell membrane, which leads to the formation of transmembrane pores and cell death due to fluid loss and disruption of the cell membrane (Lee, Chen, & Huang, 2004).However, the mechanisms by which salivary peptides execute their antibacterial activity is not well understood.

Our results showed granular patterns in the cytoplasm, pyknotic nucleoids, total lysis of bacteria, loss of membrane integrity, detachment of peptidoglycan and swollen cytoplasm with a decrease in electro‐density in *S. mutans* and *E. faecalis* incubated with the different peptides. These findings have also been observed in a previous study with *S. mutans* after the incubation with histatin 5 (Fernández‐Presas et al., 2018). Bacteria incubated with different salivary peptides exhibited pyknotic nucleoids, based on these observations, and in previous studies, we could propose that these peptides (cystatin C, histatin 5, or chromogranin A) can penetrate the cell wall and plasma membrane, accumulating within the cells and interacting with the bacterial DNA, suggesting that these peptides can lead to cell death by apoptosis, as observed under TEM. Our results agree with those obtained by Huo et al., this group studied a fragment of histatin 5, which they called (P‐113 peptide). They showed that the peptide incubated with *S. mutans* was deposited on the membrane cell and cytoplasm and concluded that the peptide can penetrate the cell wall and accumulate in the cytoplasm by binding to DNA (Huo et al., 2011).

Interestingly, salivary peptides incubated with *S. mutans* and *E. faecalis* shared similar patterns of damage, these findings enabled us to add novel information that has not been reported yet. The damage induced by the salivary peptides on the bacteria could be interpreted as a protective mechanism of innate immunity of the oral cavity. Antimicrobial peptides are natural microbicides that exhibit selective cytotoxicity against bacteria; hence, exerting minimal cytotoxicity on mammalian cells. The selective cytotoxicity of AMPs toward microbes is due to the fundamental differences in composition and structure of the host cells compared to those of pathogenic bacteria and yeasts, as well as the differential expression and localization of AMPs that prevent unwanted interactions with vulnerable host cells (Ebenhan, Gheysens, Kruger, Zeevaart, & Sathekge, 2014).

For instance, the peptides incubated with *S. mutans*, the main cariogenic agent, highlight its pathogenicity, due to its ability to form a biofilm in the oral cavity (Krzyściak, Jurczak, Kościelniak, Bystrowska, & Skalniak, 2014; Senadheera & Cvitkovitch, 2008). In turn, *E. faecalis* is the agent responsible for biofilm formation in persistent root canal infections, through the formation of multi‐layered antibiotic resistant biofilms (Blackledge, Worthington, & Melander, 2013; Dunny, Hancock, & Shankar, 2014); therefore, biofilm‐associated infections can become highly resistant to antibiotic therapy (Ike, 2017). These AMPs can inhibit biofilms and the inhibition of this process may be an effective anti‐biofilm strategy (Da Cunha et al., 2017; Sullivan et al., 2011). The results of our study demonstrate an extensive ultrastructural damage on *S. mutans* and *E. faecalis* promoted by the salivary peptides. Further experiments should be carried out using clinical isolates of these bacteria obtained from patients with oral diseases.

The study of the microbicidal mechanisms of salivary proteins and innate immunity, and the understanding of the mechanisms of action of the different antimicrobial peptides (AMPs) to combat pathogenic biofilms, could lead to potential therapeutic agents for the oral cavity. The identification of different therapeutic alternatives to contend with biofilm‐associated infections is imperative, because biofilms are partly responsible for bacterial resistance to antibiotics. The microorganisms forming biofilms are extremely difficult or impossible to eradicate (Alhede et al., 2009; Van‐G et al., 2009).

## 5 CONCLUSIONS

The present study showed that salivary peptides induce extensive damage in *E. faecalis* and *S.s mutans*. Our findings support the hypothesis that the use of antimicrobial peptides as therapeutic alternatives are valuable tools for the control of these bacteria.

## CONFLICT OF INTEREST

The authors declare no conflict of interest.

## AUTHOR CONTRIBUTIONS

Blanca Blancas and María de Lourdes Lanzagorta participate in the study design and conducted the experiments. Luis Felipe Jiménez‐Garcia and Reyna Lara prepared the electron micrographs and described the ultrastructure of the bacteria. José Luis Molinari was involved in the concept of the study and critically revised the manuscript, Ana María Fernández participated in the study design, conducted experiments, interpreted the data, drafted the manuscript and acquired the funding. All authors have read and agreed to the published version of the manuscript, (and any substantially modified version that involves the author's contribution to the study).

## Data Availability

The data that support the findings of this study are available from the corresponding author upon reasonable request.
